# Long-term safety of tiotropium/olodaterol in older patients with moderate-to-very-severe COPD in the TONADO® studies

**DOI:** 10.1038/s41533-020-00212-w

**Published:** 2020-12-04

**Authors:** Gary T. Ferguson, François Maltais, Jill Karpel, Ulrich Bothner, Isabel Kloer, Matthias Trampisch, Roland Buhl

**Affiliations:** 1 Pulmonary Research Institute of Southeast Michigan, 29255 West 10 Mile Road, Suite A, Farmington Hills, MI 48336 USA; 2 Centre de Recherche, Institut Universitaire de Cardiologie et de Pneumologie de Québec, Université Laal, 2725 Chemin Sainte-Foy, Québec City, QC G1V 4G5 Canada; 3 North Shore Medical Arts LLP, 295 Community Drive, Great Neck, New York, NY 11021 USA; 4 Boehringer Ingelheim International GmbH, Binger Strasse 173, D-55216 Ingelheim am Rhein, Germany; 5 Pulmonary Department, Johannes Gutenberg University Mainz, Langenbeckstrasse 1, D-55131 Mainz, Germany

**Keywords:** Chronic obstructive pulmonary disease, Adverse effects, Geriatrics

## Abstract

Older patients with chronic obstructive pulmonary disease (COPD) may be at increased risk of adverse events (AEs) due to decreased protective organ function and increased comorbidities. TONADO® 1 + 2 were replicate, randomized, double-blind, parallel-group, 52-week, Phase III trials comparing the efficacy and safety of tiotropium/olodaterol (5/5 µg) versus the monocomponents via the Respimat® inhaler in patients with moderate-to-very-severe COPD. In this prespecified safety analysis, patients were grouped by age. Of 3100 patients, 1585 (51.1%) were aged <65 years, 1198 (38.7%) 65–<75 years, 309 (10.0%) 75–<85 years, and eight (0.3%) ≥85 years. At baseline, 23.4% had a pre-existing cardiac disorder, 45.6% had hypertension, and 13.3% had glucose metabolism disorders, including diagnosed diabetes. Overall, there was no increase in major adverse cardiac events, other AEs, or serious AEs with tiotropium/olodaterol versus the monocomponents in any age group, supporting the safety of tiotropium/olodaterol in older patients with COPD.

## Introduction

Chronic obstructive pulmonary disease (COPD), characterized by persistent respiratory symptoms and airflow limitation, is a leading cause of morbidity and mortality worldwide^1^. The prevalence of COPD is projected to rise over the next few decades owing to the aging of the world’s population and the increased long-term exposure to risk factors for this disease^1–3^.

Long-acting bronchodilators are recommended by the Global Initiative for Chronic Obstructive Lung Disease (GOLD) guidelines for maintenance therapy of patients with moderate-to-very-severe COPD^1^. Tiotropium is an established once-daily, long-acting muscarinic antagonist (LAMA) that improves lung function and patient-reported outcomes such as dyspnea and quality of life, and reduces exacerbations in patients with COPD. Olodaterol is a more recently approved and marketed long-acting β_2_-agonist (LABA) that provides 24-h bronchodilation and symptomatic benefits in patients with COPD^4,5^. The added benefits of combining tiotropium and olodaterol as a fixed-dose combination (FDC), and the long-term efficacy and safety of tiotropium/olodaterol treatment over 52 weeks versus the monocomponents, were demonstrated in the Phase III TONADO® 1 and 2 trials^6^.

The prevalence of COPD increases with age^7^, and older age is associated with poor prognosis and an increased risk of mortality in patients with COPD, particularly in those aged 80 years and older^2^. Elderly patients with COPD may be at increased risk of adverse events (AEs) because of a general decrease in protective organ functions^8^, and an increased incidence of comorbid conditions such as cardiovascular (CV), neurologic, psychiatric, gastrointestinal, and infectious diseases. In addition, those taking multiple medications due to comorbid conditions are at increased risk of drug–drug interactions^9–11^. Therefore, it is important to determine whether the safety of COPD medications is affected by aging^12^. Inhaled therapies are recommended by GOLD for patients with COPD, irrespective of age^1^. No age-specific dose reductions of olodaterol, tiotropium, or tiotropium/olodaterol are required based on pharmacologic data^13–15^. Nevertheless, there is a need for further information on the efficacy and safety of inhaled therapies in the population of vulnerable older patients, or patients over the age of 80, as highlighted in the latest American Thoracic Society guidelines^16^.

The objectives of this analysis were to investigate the effect of age on the safety of bronchodilators and to assess the safety of tiotropium/olodaterol compared with the monocomponents in older patients with moderate-to-very-severe COPD included in the large TONADO® study population.

## Results

### Patients

Baseline characteristics are presented in Table 1. Overall, 3100 treated patients were included in this safety analysis. Of these, 1585 (51.1%) were aged <65 years, 1198 (38.7%) were aged 65–<75 years, and 309 (10.0%) were aged 75–<85 years; these were equally distributed across treatment groups. Overall, eight patients (0.3%) were aged ≥85 years (range 85–97 years); of these, three received olodaterol, four received tiotropium, and one received tiotropium/olodaterol (Table 1). Study drug exposure was similar among patients aged <65, 65–<75, and 75–<85 years in all treatment groups, with no age-related increase or decrease observed (Table 1). At baseline, the proportion of current smokers decreased with increasing age; there was a similar distribution of smokers and ex-smokers across treatment groups (Table 1).

**Table 1:** Demographic and baseline patient characteristics by age category.

Characteristic	Olodaterol (*n* = 1038)^a^			Tiotropium (*n* = 1033)^b^			Tiotropium/olodaterol (*n* = 1029)^c^		
	Age (years)			Age (years)			Age (years)		
	<65	65–<75	75–<85	<65	65–<75	75–<85	<65	65–<75	75–<85
Treated patients, *n* (%)	520 (50.1)	408 (39.3)	107 (10.3)	540 (52.3)	383 (37.1)	106 (10.3)	525 (51.0)	407 (39.6)	96 (9.3)
Male, *n* (%)	361 (69.4)	312 (76.5)	89 (83.2)	363 (67.2)	299 (78.1)	89 (84.0)	354 (67.4)	301 (74.0)	77 (80.2)
Age, years	57.6 ± 5.2	68.9 ± 2.8	77.6 ± 2.0	57.3 ± 5.4	69.1 ± 2.8	77.4 ± 2.3	57.2 ± 0.4	69 ± 2.7	77.6 ± 2.3
BMI, kg/m^2^	26 ± 6.4	25.8 ± 5.0	25.5 ± 5.2	26.2 ± 5.7	25.8 ± 4.8	25.1 ± 4.9	26.2 ± 6.0	25.5 ± 4.7	24.8 ± 5.0
Smoking history, *n* (%)									
Current smoker	260 (50.0)	97 (23.8)	21 (19.6)	266 (49.3)	91 (23.8)	13 (12.3)	268 (51.0)	113 (27.8)	19 (19.8)
Ex-smoker	260 (50.0)	311 (76.2)	86 (80.4)	274 (50.7)	292 (76.2)	93 (87.7)	257 (49.0)	294 (72.2)	77 (80.2)
Pack-years	42.5 ± 21.4	51.1 ± 27.3	54.3 ± 29.6	42.1 ± 23.2	50.2 ± 29.3	53.3 ± 37.3	43.9 ± 23.3	48.9 ± 24.7	53.0 ± 37.0
GOLD stage, *n* (%)									
GOLD 2	255 (49.0)	215 (52.7)	60 (56.1)	278 (51.5)	184 (48.0)	52 (49.1)	245 (46.7)	198 (48.6)	58 (60.4)
GOLD 3	191 (36.7)	143 (35.0)	43 (40.2)	192 (35.6)	151 (39.4)	43 (40.6)	211 (40.2)	162 (39.8)	35 (36.5)
GOLD 4	74 (14.2)	50 (12.3)	4 (3.7)	69 (12.8)	48 (12.5)	11 (10.4)	69 (13.1)	47 (11.5)	3 (3.1)
Baseline pulmonary medication, *n* (%)	409 (78.7)	334 (81.9)	91 (85.0)	398 (73.7)	315 (82.2)	86 (81.1)	402 (76.6)	333 (81.8)	84 (87.5)
LAMA (tiotropium)	159 (30.6)	166 (40.7)	39 (36.4)	155 (28.7)	148 (38.6)	42 (39.6)	171 (32.6)	154 (37.8)	53 (55.2)
SAMA	71 (13.7)	52 (12.7)	11 (10.3)	76 (14.1)	46 (12.0)	9 (8.5)	77 (14.7)	36 (8.8)	12 (12.5)
LABA^d^	236 (45.4)	202 (49.5)	51 (47.7)	212 (39.3)	190 (49.6)	47 (44.3)	244 (46.5)	192 (47.2)	49 (51.0)
SABA	232 (44.6)	158 (38.7)	32 (29.9)	218 (40.4)	144 (37.6)	38 (35.8)	219 (41.7)	145 (35.6)	36 (37.5)
ICS	241 (46.3)	205 (50.2)	57 (53.3)	226 (41.9)	187 (48.8)	52 (49.1)	246 (46.9)	213 (52.3)	46 (47.9)
Xanthines	40 (7.7)	36 (8.8)	19 (17.8)	52 (9.6)	44 (11.5)	13 (12.3)	40 (7.6)	57 (14.0)	11 (11.5)
Baseline cardiovascular medication, *n* (%)	272 (52.3)	270 (66.2)	75 (70.1)	280 (51.9)	237 (61.9)	77 (72.6)	259 (49.3)	263 (64.6)	58 (60.4)
Study exposure, days	331.6 ± 89.6	322.7 ± 102.1	316.5 ± 104.1	332.3 ± 92.9	330.2 ± 91.6	333.5 ± 83.7	345.2 ± 73.1	336 ± 82.8	328.3 ± 96.8

Patient demographics previously published in Buhl et al.^18^.

*BMI* body mass index, *GOLD* Global Initiative for Chronic Obstructive Lung Disease, *ICS* inhaled corticosteroid, *LABA* long-acting β_2_-agonist, *LAMA* long-acting muscarinic antagonist, *SABA* short-acting β_2_-agonist, *SAMA* short-acting muscarinic antagonist.

^a^Includes three patients aged ≥85 years.

^b^Includes four patients aged ≥85 years.

^c^Includes one patient aged ≥85 years.

^d^The use of a LABA in combination with an inhaled steroid was counted under both “LABA” and “ICS”. Values are presented as mean ± standard deviation unless otherwise stated.

In the total treated population included in this analysis, 76.3% of patients aged <65 years were receiving pulmonary medication at baseline. This increased to 82.0% in patients aged 65–<75 years and to 84.5% in those aged 75–<85 years. LABAs and inhaled steroids were used at baseline by 43.7% and 45.0% of patients aged <65 years, by 48.7% and 50.5% patients aged 65–<75 years, and by 47.6% and 50.2% of those aged 75–<85 years. The proportion of patients receiving tiotropium also increased with age.

The proportion of patients with the most severe airflow obstruction, GOLD 4, appeared to decrease with increasing age.

**Fig. 1:**
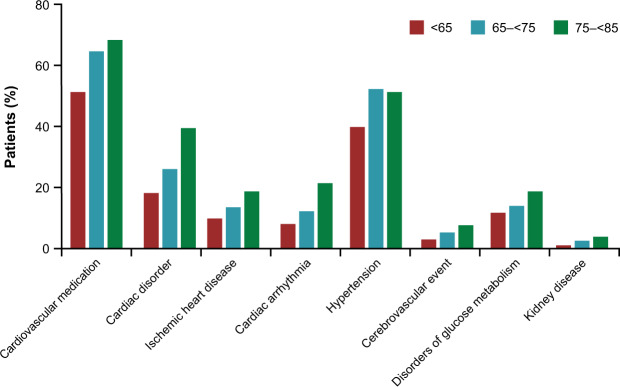
Baseline comorbidity by age category. Proportion of patients in each age category (<65 years old; 65–≤75 years old; >75 years old) with comorbidities at baseline.

Comorbidity at baseline was common in patients participating in TONADO®, as expected in a population of elderly patients with COPD. Among the total treated population included in this analysis, almost one-quarter (23.4%) of patients had a pre-existing cardiac disorder (defined using Medical Dictionary for Regulatory Activities [MedDRA] version 16.1 system organ class [SOC]), 45.6% had hypertension, and 13.3% had disorders of glucose metabolism, including diagnosed diabetes. As expected, the proportion of patients with comorbidity increased with advancing age (Fig. 1). In particular, an increase in age resulted in a progressive increase in the diagnosis of CV diseases and hypertensive diseases.

### Summary of safety outcomes

**Fig. 2:**
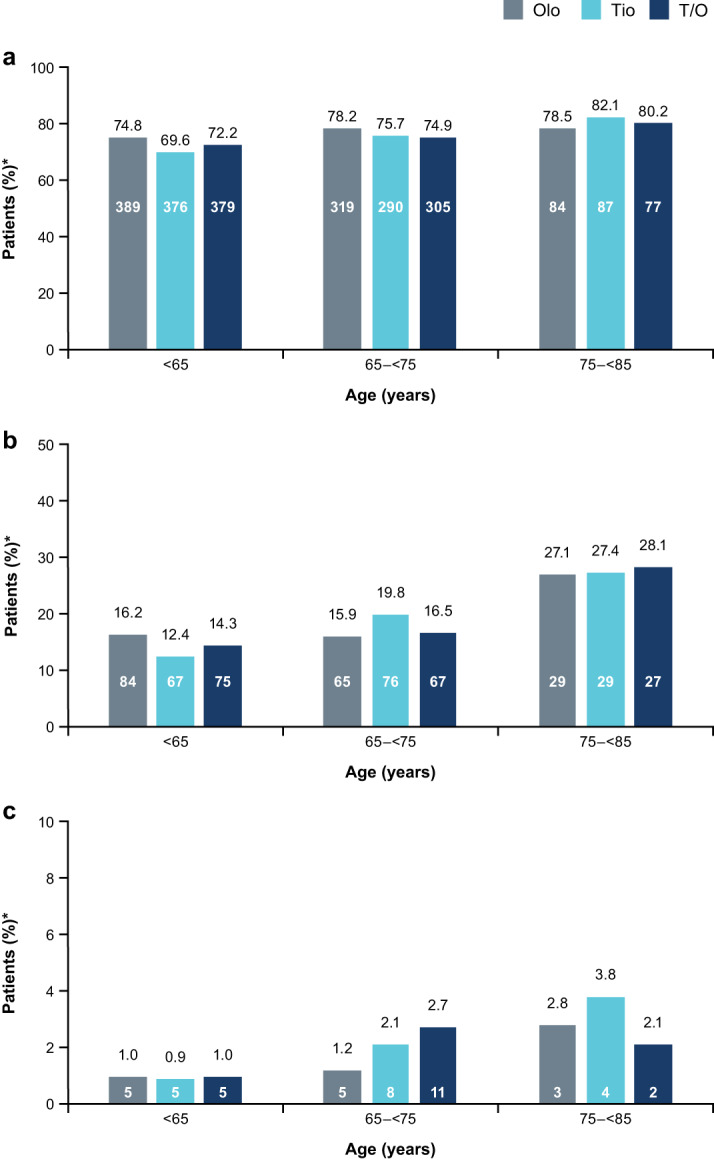
Incidence of adverse events and serious adverse events by age category. **a** Patients with any AE, **b** patients with an SAE, and **c** patients with a fatal SAE. *Patient numbers within bars, and percentage of the total number of patients above bars; ^†^no significant difference between treatment groups in any age category (Fisher’s exact test: *p* > 0.05). *AE* adverse event, *Olo* olodaterol, *SAE* serious adverse event, *T/O* tiotropium/olodaterol, *Tio* tiotropium. Data from Fig. 2a and b have previously been published in Ferguson et al.^17^ as a table.

In this study population, the proportion of patients who reported AEs, particularly serious AEs (SAEs) and fatal SAEs, was higher in older patients (Fig. 2 and Table 2). Data in Fig. 2a and b were previously reported in Ferguson et al.^17^; however, the present analysis includes data on fatal SAEs and also provides statistical comparisons between treatment groups within each age category. Within each age group, there were no significant differences in the proportion of patients reporting AEs, SAEs, or fatal SAEs between the treatment arms.

**Table 2:** Summary of adverse events by age category and treatment group.

Patients, *N* (%)	Olodaterol 5 μg (*n* = 1035)			Tiotropium 5 μg (*n* = 1029)			Tiotropium/olodaterol 5/5 μg (*n* = 1028)			Total (*N* = 3092)		
	Age (years)			Age (years)			Age (years)			Age (years)		
	<65	65–<75	75–<85	<65	65–<75	75–<85	<65	65–<75	75–<85	<65	65–<75	75–<85
Total treated	520 (100)	408 (100)	107 (100)	540 (100)	383 (100)	106 (100)	525 (100)	407 (100)	96 (100)	1585 (100)	1198 (100)	309 (100)
Any AEs	389 (74.8)	319 (78.2)	84 (78.5)	376 (69.6)	290 (75.7)	87 (82.1)	379 (72.2)	305 (74.9)	77 (80.2)	1144 (72.2)	914 (76.3)	248 (80.3)
SAEs^a^	84 (16.2)	65 (15.9)	29 (27.1)	67 (12.4)	76 (19.8)	29 (27.4)	75 (14.3)	67 (16.5)	27 (28.1)	226 (14.3)	208 (17.4)	85 (27.5)
Fatal	5 (1.0)	5 (1.2)	3 (2.8)	5 (0.9)	8 (2.1)	4 (3.8)	5 (1.0)	11 (2.7)	2 (2.1)	15 (0.9)	24 (2.0)	9 (2.9)
Life-threatening	2 (0.4)	0 (0.0)	0 (0.0)	1 (0.2)	0 (0.0)	1 (0.9)	2 (0.4)	1 (0.2)	2 (2.1)	5 (0.3)	1 (0.1)	3 (1.0)
Disability	0 (0.0)	1 (0.2)	0 (0.0)	1 (0.2)	0 (0.0)	1 (0.9)	2 (0.4)	0 (0.0)	1 (1.0)	3 (0.2)	1 (0.1)	2 (0.6)
Requires hospitalization	77 (14.8)	56 (13.7)	26 (24.3)	62 (11.5)	72 (18.8)	21 (19.8)	69 (13.1)	58 (14.3)	26 (27.1)	208 (13.1)	186 (15.5)	73 (23.6)
Prolonged hospitalization	3 (0.6)	7 (1.7)	2 (1.9)	1 (0.2)	1 (0.3)	1 (0.9)	3 (0.6)	3 (0.7)	0 (0.0)	7 (0.4)	11 (0.9)	3 (1.0)
Other	8 (1.5)	7 (1.7)	4 (3.7)	3 (0.6)	8 (2.1)	7 (6.6)	5 (1.0)	5 (1.2)	2 (2.1)	16 (1.0)	20 (1.7)	13 (4.2)

*AE* adverse event, *MedDRA* Medical Dictionary for Regulatory Activities, *SAE* serious adverse event.

^a^A patient may be counted in >1 seriousness criterion. Percentages were calculated using the total number of patients per treatment as the denominator. MedDRA version 16.1 used for AE reporting.

**Fig. 3:**
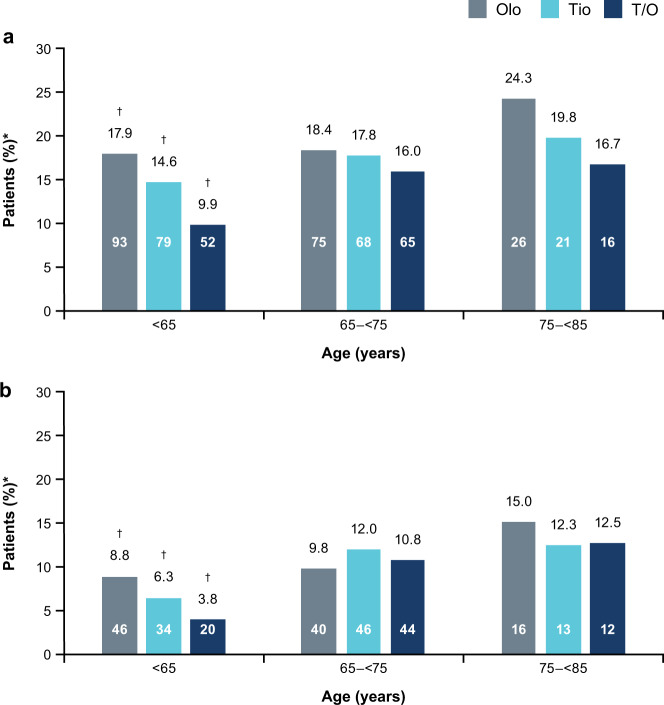
Discontinuation of study medication and study participation by age category. **a** Patients who discontinued trial medication or **b** patients who discontinued due to an AE. *Patient numbers within bars, and percentage of the total number of patients above bars; ^†^significant treatment differences in the <65-years group (Fisher’s exact test: *p* < 0.05), but no significant treatment differences for any other age group. *AE* adverse event, *Olo* olodaterol, *T/O* tiotropium/olodaterol, *Tio* tiotropium.

Similarly, a greater proportion of older patients appeared to discontinue treatment because of an AE (Fig. 3). Discontinuations, including discontinuations due to an AE, were either similar or lower with tiotropium/olodaterol compared with the monocomponents, across age categories (Fig. 3). In the <65-years group, discontinuation was significantly lower in the tiotropium/olodaterol treatment arm compared with the monocomponents (prematurely discontinued trial medication *p* < 0.001; discontinued due to an AE *p* < 0.01).

### Safety outcomes in individual patients aged ≥ 85 years

Eight patients were aged ≥85 years, and we have summarized the SAEs experienced by these patients in Table 3. Of AEs occurring in patients aged ≥85 years receiving olodaterol, one was fatal (cerebrovascular infarction), and three required hospitalization (failure to thrive, paroxysmal atrial fibrillation, and cellulitis of the leg) (Table 3). None of the AEs reported in patients in this age group who received tiotropium or tiotropium/olodaterol led to discontinuation of the study drug. The oldest patient included in this analysis—a 97-year-old male—received tiotropium and experienced two AEs during the study period (angiopathy and bruising of the arm and face, and lower limb wounds). Neither of these were considered serious, and the patient subsequently recovered and remained on treatment (Table 3).

**Table 3:** Adverse events in individual patients aged ≥85 years (*n* = 8) with tiotropium/olodaterol versus tiotropium or olodaterol alone.

Treatment group	Age	Sex	Event (LLT)	Start day^a^	Duration (days)		Study medication	Outcome	Serious
Olodaterol 5 μg	85	M	Cerebrovascular infarction	26	27		Discontinued	Fatal	Fatal
			Subdural hematoma	26	5		Discontinued	Recovered	Life-threatening
			Left deep vein thrombosis	30	16		–	Recovered	Other
			Fall, fractured nose	37	16		–	Recovered	N
			Depression	37	–		–	Unknown	N
			Poor swallowing reflex	38	15		–	Recovered with sequelae	N
			Failure to thrive	42	–		–	Unknown	Hospitalization
	86	F	COPD exacerbation	18	8		Continued	Recovered	N
			Fall, thorax pain	46	82		Continued	Recovered	N
			Headache	76	3		Continued	Recovered	N
			Paroxysmal atrial fibrillation	157	5		Continued	Recovered	Hospitalization
			COPD exacerbation	245	10		Continued	Recovered	N
	87	M	Cellulitis of leg	139	88		Continued	Recovered	Hospitalization
			COPD exacerbation	173	11		Continued	Recovered	N
			Chest pain	213	3		Continued	Recovered	N
Tiotropium 5 μg	89	M	Bleeding nose	161	1		Continued	Recovered	N
			Common cold	294	15		Continued	Recovered	N
	97	M	Angiopathy	43	43		Continued	Recovered	N
			Bruising of arm/face; lower limb wound	285	43		Continued	Recovered	N
	86	M	Laceration of head	38	7		Continued	Recovered	N
			Sore throat	116	5		Continued	Recovered	N
	86	M	Impacted cerumen	79	23		Continued	Recovered	N
			Upper respiratory tract infection	112	18		Continued	Recovered	N
			Dry mouth	305	–		Continued	Not recovered	N
Tiotropium/olodaterol 5/5 μg	85	M	None reported	–	–		–	–	–

*COPD* chronic obstructive pulmonary disease, *F* female, *LLT* Medical Dictionary for Regulatory Activities low-level term, *M* male, *N* no, *Y* yes.

^a^After start of randomized study medication.

### Clinically relevant AEs associated with advancing age

**Fig. 4:**
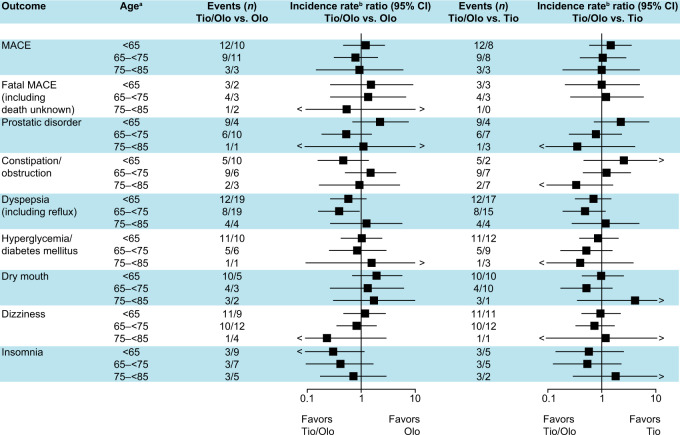
Exposure-adjusted incidence rate ratios and 95% confidence intervals of clinically relevant adverse event groups associated with advancing age comparing tiotropium/olodaterol with the monocomponents. The overall percentage of events was <1%. ^a^≥85 years not displayed due to low patient numbers (*n* = 8); ^b^treatment exposure time adjusted. *CI* confidence interval, *MACE* major adverse cardiovascular event, *Olo* olodaterol, *Tio* tiotropium.

The number of patients with a clinically relevant AE that may be associated with increasing age and deemed a burden in older patients was similar with tiotropium/olodaterol and with the monocomponents, irrespective of age (Fig. 4 and Supplementary Fig. 1). In general, there was no significant difference in the exposure-adjusted incidence rates for CV, respiratory, and age-related AEs between the treatment groups or between age categories. Overall, the safety profile of tiotropium/olodaterol in subgroups of patients with increasing age was comparable with that of the monocomponents and consistent with the results of the overall analysis.

## Discussion

Consistent with real-world populations of patients with COPD, the TONADO® studies included a considerable proportion of elderly patients with pre-existing disease and comorbidity. Our analysis showed that, as expected with advancing age, more comorbidities were reported and AEs and SAEs occurred more frequently within the older age categories. However, we found no increase in treatment discontinuations or age-associated clinically relevant AEs with tiotropium/olodaterol compared with the monocomponents in any age group. Furthermore, the safety profiles of tiotropium/olodaterol and its monocomponents within the analyzed subgroups were consistent with the results of the overall study safety analysis^6^, supporting the safety of tiotropium/olodaterol combination treatment among all age groups analyzed. Previously published data from TONADO®^18^ and other Phase III trials^17,19^ showed that tiotropium/olodaterol displayed similar efficacy and safety profiles to the monocomponents, even in older patients. Our findings are consistent with these studies and provide further safety information, including AEs in patients over 85 years as well as AEs leading to discontinuation from the TONADO® trials stratified by age group. The proportion of patients with the most severe airflow obstruction (GOLD 4) appeared to decrease with increasing age, which could potentially be due to survivor bias.

The global prevalence of COPD increased from 10.7% (7.3%–14.0%) in 1990 to 11.7% (8.4%–15.0%) in 2010, with a higher prevalence observed among subgroups of patients with advancing age^20^. With the aging of the world’s population, the global prevalence of COPD is projected to rise; accordingly, the World Health Organization predicts that COPD will be the third leading cause of death worldwide by 2030^21^. As COPD carries a worse prognosis in older patients, this increase will impact the overall burden of COPD in terms of morbidity, mortality, and healthcare costs^2^. In the present analysis, the proportion of patients experiencing AEs or SAEs increased with advancing age, consistent with previous findings in this patient population^17^. The TIOSPIR analysis, which compared the safety profile of the Respimat® inhaler with that of the HandiHaler inhaler, identified similar safety profiles between these two devices^22^.

Over 80% of older patients with COPD are reported to have comorbidities^11,23^. Our observation of an increasing prevalence of comorbidities, including CV comorbidities, in patients with advancing age in the TONADO® studies, is consistent with previous reports. CV comorbidities are common among patients with COPD, particularly in older individuals; indeed, CV events, including heart failure and myocardial infarction, are common causes of hospitalization among elderly patients with COPD^24^. Additionally, a link has been established between COPD and CV disease^24^. Thus, the results of the present analysis are reassuring, since no increase in the proportion of patients with major adverse CV events (MACEs) was observed with tiotropium/olodaterol and advancing age compared with the monocomponents, indicating that there were no added safety concerns with dual bronchodilation compared with the monocomponents. In TONADO®, around 11% of patients were prescribed a β-blocker; data from a post hoc analysis indicated no safety concerns associated with concomitant use of tiotropium/olodaterol and β-blockers^25^. However, results may have been affected by the higher mean baseline post-bronchodilator forced expiratory volume in 1 s (FEV_1_) in the β-blocker group and the fact that the study did not include patients with significant cardiac disease^25,26^. In any case, the benefits of β-blockers in older patients with COPD and coexistent CV morbidities remain unclear^27^.

Physiologic, anatomical, and immunologic changes that occur during aging, including the functional deterioration of tissues and organs, impact the management of chronic conditions such as COPD^28^. In addition, age-related changes in lung, liver, and kidney function may alter with the pharmacokinetics of inhaled bronchodilators used in the treatment of chronic respiratory conditions^8,9^. As outlined by Laforce et al., comparable safety and tolerability of tiotropium/olodaterol were demonstrated relative to its monocomponents, irrespective of renal impairment^29^. However, caution should be taken when prescribing in elderly patients due to the risk of deteriorating renal impairment. Patient age had a small effect on systemic exposure to combination umeclidinium/vilanterol FDC or the monocomponents. A 10% increase in age was associated with a 7% and 4% increase in the apparent inhaled clearance of umeclidinium and vilanterol, respectively^30^. Age was also found to affect the inhaled clearance of vilanterol in patients with COPD, although the magnitude of the responses was not sufficient to warrant dose adjustments in this patient population^31^. Despite small changes in the pharmacokinetics of bronchodilator drugs in elderly patients with COPD, these changes did not translate into a need for dose adjustments in an older population. Accordingly, no dose adjustments are recommended for olodaterol or tiotropium monotherapy, for combination therapy, or for other LAMA/LABA combinations, including indacaterol maleate/glycopyrronium, umeclidinium/vilanterol, and aclidinium/formoterol in elderly patients with COPD^14,15,32–35^.

Age-related changes in receptor populations and nervous control mechanisms within the airways may interfere with the efficacy of pharmacologic treatments, such as bronchodilators. Older individuals require lower doses of methacholine to initiate bronchoconstriction, signifying increased bronchial reactivity^36^. However, bronchodilator responsiveness may decline with increasing age, as shown by a longer onset of bronchodilator action in this population, potentially due to age-related dysfunction in β_2_-receptor activity within the airways^8,36^. Data from human studies on potential age-related changes in airway muscarinic receptors are limited, and their clinical significance in older patients has not yet been identified^8^. Thus, the efficacy of tiotropium in patients aged ≥80 years was shown to be comparable with that in patients aged <80 years, with a similar safety profile across the age groups^37^. Additionally, tiotropium/olodaterol demonstrated significant improvements from baseline in lung function and symptomatic response across patients of all age groups in a subgroup analysis of the TONADO® and OTEMTO® studies. Importantly, no safety concerns were found in elderly patients receiving tiotropium/olodaterol versus the monocomponents or placebo^17^.

Side effects associated with the use of β_2_-agonists increase dose-proportionally and may increase with advancing age, particularly with the concomitant use of other drugs in patients with comorbidities, such as the elderly. The most common side effect associated with the use of anticholinergics is dry mouth^38^, which in elderly patients can impact the ability to communicate, and may lead to secondary events such as predisposing to malnutrition and increasing the risk of respiratory infections. Nevertheless, in the present analysis, no increase in the proportion of patients experiencing dry mouth was observed with tiotropium/olodaterol compared with the monocomponents.

A strength of this analysis is that the large size of the TONADO® studies permits the analysis of older patients, including patients up to 97 years of age. Limitations include the exclusion of patients with very severe or unstable conditions from the TONADO® studies; therefore, these data may not be representative of elderly patients with extreme individual conditions encountered in clinical practice. Also, due to a low number of patients in the oldest age group (>85 years), this group was too small for statistical analysis. Additionally, as this is a post hoc safety analysis, these results are not confirmatory.

The results of the subgroup analyses are consistent with those of the overall analysis, indicating that tiotropium/olodaterol is as safe and as well tolerated as its monocomponents in patients with advancing age and comorbidities^17^. The TONADO® studies demonstrated benefits of tiotropium/olodaterol combination therapy over tiotropium or olodaterol alone on improvements in lung function and health-related quality of life over 1 year in patients with moderate-to-severe COPD, with a comparable incidence of AEs between groups^6^. In combination with data showing that combination therapy is efficacious in all patients stratified by age group^17^, our data show that treating elderly patients with combination therapy offers a favorable benefit–risk ratio, with benefits in efficacy observed without additional safety concerns compared with those observed with monotherapy treatment.

In conclusion, the TONADO® studies compared the efficacy and safety of tiotropium/olodaterol combination and the monocomponents in patients with moderate-to-severe COPD^6^. As expected, the patient population evaluated in the present analysis included a considerable proportion of elderly patients with pre-existing disease and comorbidity, which is consistent with the findings of previous studies and with real-world clinical experience. The proportion of patients with any AE increased with age, with no differential increase in clinically relevant age-related AEs when comparing tiotropium/olodaterol with the monocomponents. These findings should reassure clinicians that tiotropium/olodaterol combination treatment is as safe and well tolerated as the monotherapies in older patients with COPD. The results of this analysis further support the current GOLD recommendation that long-acting bronchodilators alone and in combination are the basis of COPD treatment^1^.

## Methods

### Study design

The TONADO® studies (1237.5 [NCT01431274] and 1237.6 [NCT01431287]) were two replicate, randomized, double-blind, parallel-group, 52-week, Phase III trials that compared tiotropium/olodaterol FDC (2.5/5 and 5/5 μg) with the monocomponents tiotropium (2.5 and 5 μg) and olodaterol (5 μg), all via the Respimat^®^ inhaler, in patients with moderate-to-very-severe COPD^6^. Main inclusion criteria were: outpatients aged ≥40 years with a history of moderate-to-very-severe COPD (GOLD Stage 2–4); post-bronchodilator FEV_1_ < 80% of predicted normal; post-bronchodilator FEV_1_/forced vital capacity <70%; and current or ex-smokers with a smoking history of >10 pack-years. Patients were excluded if they had a significant disease other than COPD. There was no exclusion criterion based on older age; full inclusion and exclusion criteria have been described in detail elsewhere^6^. All patients provided written informed consent prior to inclusion. The protocols of the TONADO® studies were approved by the authorities and the ethics committees of the respective institutions.

### Evaluations and outcome measures

In this prespecified safety analysis of pooled data from the TONADO® studies, patient age cohorts were defined as follows: <65; 65–<75; 75–<85; and ≥85 years. Because of the small number of patients aged ≥85 years (*n* = 8), they are not included in the stratified comparative analysis and are described case by case in the text.

Investigator-reported treatment-emergent AEs (TEAEs) were pooled from both TONADO® studies, in which all AEs and SAEs occurring from consent through 21 days after the last dose of study medication were collected. An SAE was defined as any AE that either resulted in death, was immediately life-threatening, resulted in persistent or significant disability or incapacity, required hospitalization, prolonged an existing hospitalization, resulted in a congenital anomaly/birth defect, or had any other reason representing a significant hazard. All deaths and all SAEs were adjudicated by an adjudication committee comprising international expert clinicians experienced in pulmonology and cardiology, who were independent of the trial conduct and sponsor, and blinded to the study medication treatment. All AEs shown are TEAEs; these were defined as those that started after the first dose of study medication and up to 21 days after the last intake of study medication.

### Analysis

AEs were coded as per the MedDRA version 16.1 and assigned to SOCs. A composite endpoint of MACEs was used. This included fatal AEs in the cardiac disorder and vascular disorder SOCs, with fatal and non-fatal myocardial infarction, fatal and non-fatal stroke, sudden death, sudden cardiac death, and cardiac death.

Here, results for tiotropium/olodaterol 5/5 μg, tiotropium 5 μg, and olodaterol 5 μg are presented, as these doses represent the experience for the marketed products.

Two-tailed Fisher’s exact tests were used to test for differences between treatments in the frequency of AEs and discontinuations in each age group.

### Reporting summary

Further information on research design is available in the Nature Research Reporting Summary linked to this article.

## Supplementary information

Supplementary information

Reporting summary

## Data Availability

Data used in this analysis are available from the corresponding author on reasonable request.
