# Supplementary material for: Long-term safety of tiotropium/olodaterol in older patients with moderate-to-very-severe COPD in the TONADO® studies
Source: NPJ Prim Care Respir Med. 2020 Dec 4;30:53. doi: 10.1038/s41533-020-00212-w (PMC7719164; doi:10.1038/s41533-020-00212-w)
Supplement: Supplementary file 1 — Supplementary information [file 41533_2020_212_MOESM1_ESM.pdf]

Supplementary Data

Long-term safety of tiotropium/olodaterol in older patients with moderate-to-very-severe COPD: an analysis of the TONADO® studies

Gary T. Ferguson, François Maltais, Jill Karpel, Ulrich Bothner, Isabel Kloer, Matthias Trampisch, Roland Buhl

Supplementary Figure 1: Exposure-adjusted incidence rate ratios and 95% confidence intervals (forest plots) of clinically relevant adverse events associated with increasing age comparing tiotropium/olodaterol with the monocomponents

(a) Cardiovascular events

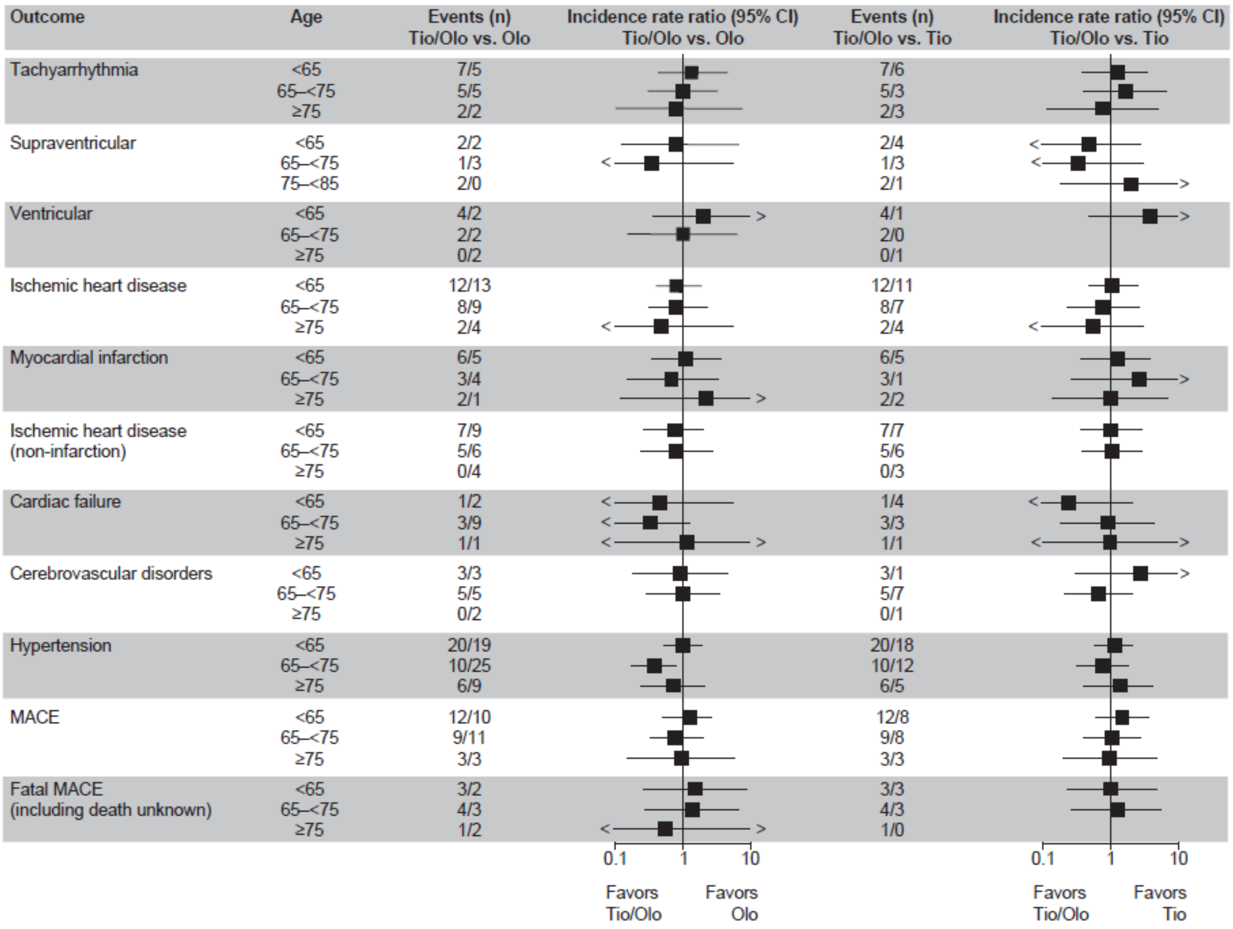

(b) Respiratory events

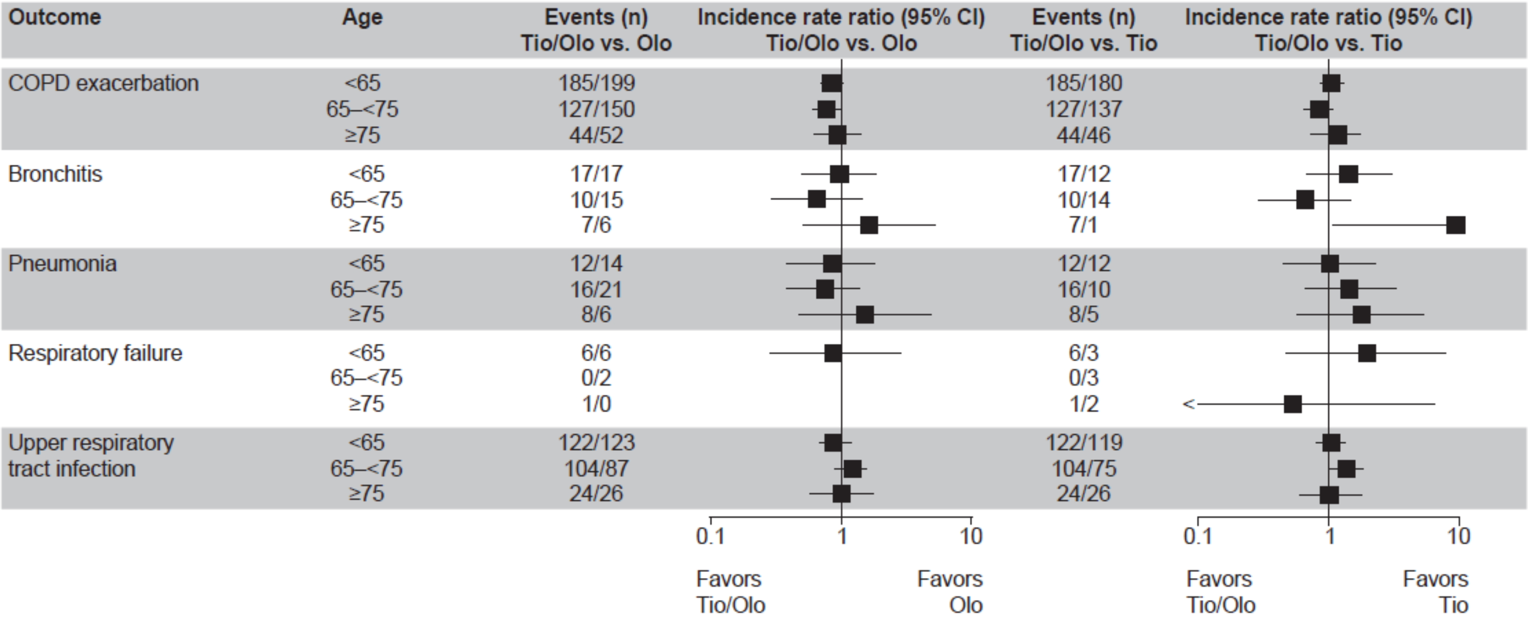

(c) Old age-related events

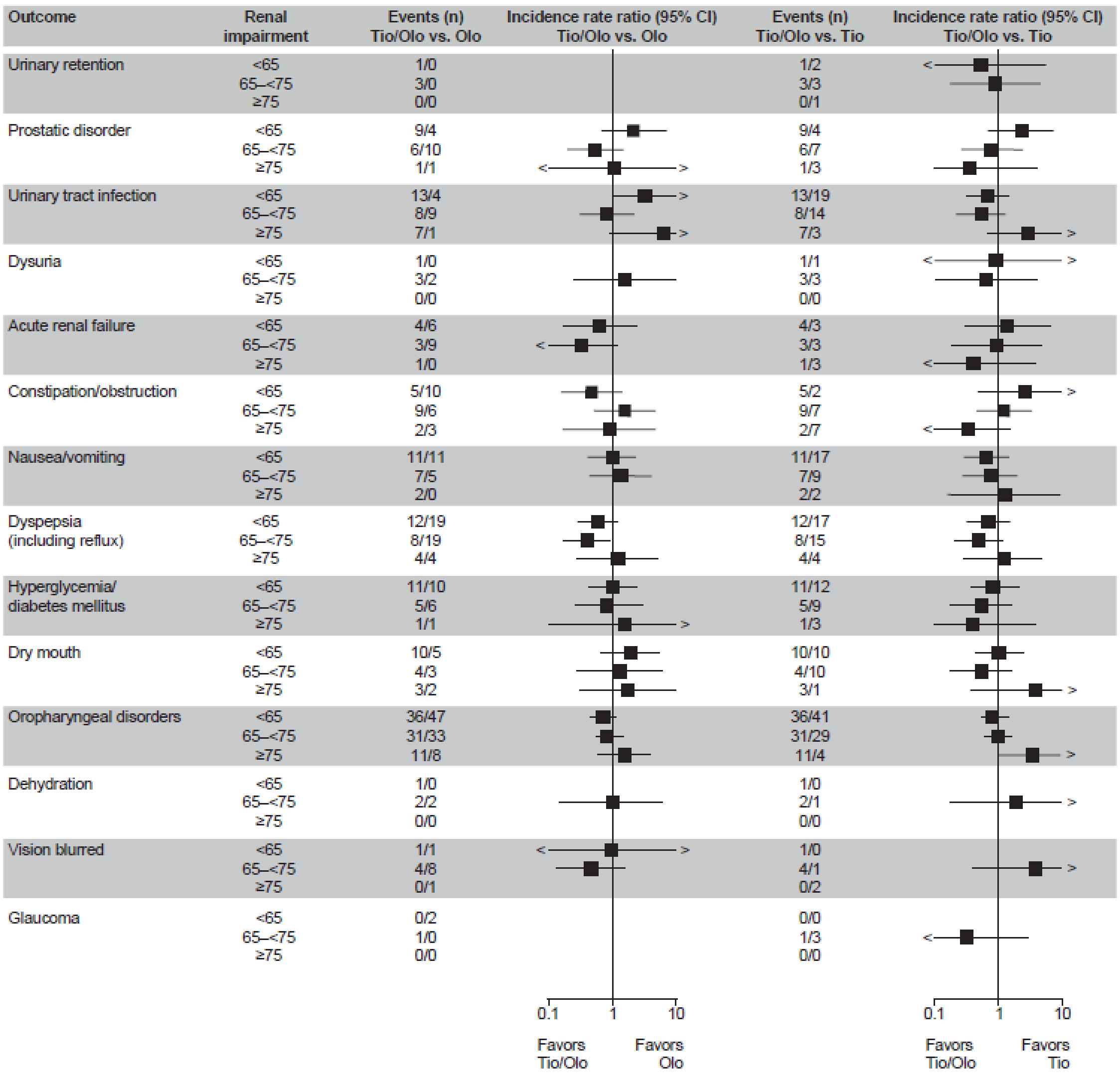

Outcome	Renal impairment	Events (n) Tio/Olo vs. Olo	Events (n) Tio/Olo vs. Tio
Urinary retention	<65	1/0	1/2
	65–<75	3/0	3/3
	≥75	0/0	0/1
Prostatic disorder	<65	9/4	9/4
	65–<75	6/10	6/7
	≥75	1/1	1/3
Urinary tract infection	<65	13/4	13/19
	65–<75	8/9	8/14
	≥75	7/1	7/3
Dysuria	<65	1/0	1/1
	65–<75	3/2	3/3
	≥75	0/0	0/0
Acute renal failure	<65	4/6	4/3
	65–<75	3/9	3/3
	≥75	1/0	1/3
Constipation/obstruction	<65	5/10	5/2
	65–<75	9/6	9/7
	≥75	2/3	2/7
Nausea/vomiting	<65	11/11	11/17
	65–<75	7/5	7/9
	≥75	2/0	2/2
Dyspepsia (including reflux)	<65	12/19	12/17
	65–<75	8/19	8/15
	≥75	4/4	4/4
Hyperglycemia/ diabetes mellitus	<65	11/10	11/12
	65–<75	5/6	5/9
	≥75	1/1	1/3
Dry mouth	<65	10/5	10/10
	65–<75	4/3	4/10
	≥75	3/2	3/1
Oropharyngeal disorders	<65	36/47	36/41
	65–<75	31/33	31/29
	≥75	11/8	11/4
Dehydration	<65	1/0	1/0
	65–<75	2/2	2/1
	≥75	0/0	0/0
Vision blurred	<65	1/1	1/0
	65–<75	4/8	4/1
	≥75	0/1	0/2
Glaucoma	<65	0/2	0/0
	65–<75	1/0	1/3
	≥75	0/0	0/0

Incidence rate ratio (95% CI) Tio/Olo vs. Olo; Incidence rate ratio (95% CI) Tio/Olo vs. Tio

(c) Old age-related events (cont.)

Outcome	Renal impairment	Events (n) Tio/Olo vs. Olo	Incidence rate ratio (95% CI) Tio/Olo vs. Olo	Events (n) Tio/Olo vs. Tio	Incidence rate ratio (95% CI) Tio/Olo vs. Tio
Dizziness	<65	11/9		11/11	
	65–<75	10/12		10/12	
	≥75	1/4		1/1	
Restlessness/agitation	<65	0/1		0/1	
	65–<75	0/0		0/0	
	≥75	0/0		0/0	
Anxiety	<65	4/6		4/8	
	65–<75	1/8		1/0	
	≥75	0/1		0/0	
Asthenia	<65	8/9		8/8	
	65–<75	6/3		6/5	
	≥75	1/5		1/0	
Insomnia	<65	3/9		3/5	
	65–<75	3/7		3/5	
	≥75	3/5		3/2	
Dementia	<65	2/3		2/4	
	65–<75	2/2		2/3	
	≥75	2/2		2/0	
Confusion	<65	0/1		0/2	
	65–<75	1/2		1/0	
	≥75	1/1		1/0	
Depression	<65	7/6		7/6	
	65–<75	5/1		5/2	
	≥75	2/1		2/0	
Tremor	<65	1/0		1/1	
	65–<75	6/2		6/2	
	≥75	1/0		1/0	
Muscle spasms	<65	4/9		4/4	
	65–<75	5/3		5/3	
	≥75	0/4		0/1	
Accidents and injuries (including fall)	<65	40/35		40/33	
	65–<75	34/23		34/24	
	≥75	11/8		11/9	

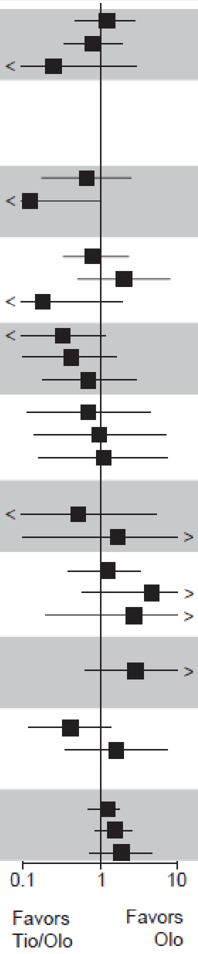

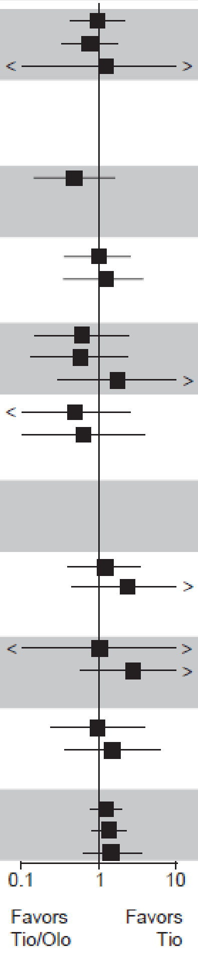

Abbreviations: CI=confidence interval; MACE=major adverse cardiovascular event; Olo=olodaterol; Tio=tiotropium
